# Health status and health care utilization after discharge from geriatric in-hospital stay – description of a register-based study

**DOI:** 10.1186/s12913-021-06751-3

**Published:** 2021-07-31

**Authors:** E. Rydwik, R. Lindqvist, C. Willers, L. Carlsson, G. H. Nilsson, A. Lager, M. Dreilich, A. Lindh Mazya, T. Karlsson, H. Alinaghizadeh, A-M Boström

**Affiliations:** 1 Department of Neurobiology, Care Sciences and Society, Division of Physiotherapy, Karolinska Institutet, Alfred Nobels Allé 23, 141 83 Huddinge, Sweden; 2 Stockholm Region Council, FOU nu, Research and Development Center for the Elderly, Järfälla, Sweden; 3 Women’s Health and Allied Health Professionals Theme, Medical Unit Occupational Therapy and Physiotherapy, Karolinska University Hospital, Solna, Sweden; 4 Department of Learning, Informatics, Management, and Ethics (LIME), Division of Innovative Care Research, Karolinska Institutet, Solna, Sweden; 5 Department of Neurobiology, Care Sciences and Society, Division of Family Medicine and Primary care, Karolinska Institutet, Huddinge, Sweden; 6 Stockholm Region Council, Academic Primary Care Center, Stockholm, Sweden; 7 Stockholm Region Council, Center for Epidemiology and Society, Stockholm, Sweden; 8 Advanced Home Care, Familjeläkarna, Stockholm, Sweden; 9 Department of Neurobiology, Care Sciences and Society, Division of Departmental Geriatrics, Karolinska Institutet, Huddinge, Sweden; 10 Geriatric Department, Danderyd Hospital, Danderyd, Sweden; 11 Department of Neurobiology, Care Sciences and Society, Division of Nursing, Karolinska Institutet, Huddinge, Sweden; 12 Inflammation and Aging Theme, Karolinska University Hospital, Huddinge, Sweden; 13 Stockholms Sjukhem, R&D unit, Stockholm, Sweden

**Keywords:** Care process, Re-admission, Multi-morbidity, Disability

## Abstract

**Background:**

This study is the first part of a register-based research program with the overall aim to increase the knowledge of the health status among geriatric patients and to identify risk factors for readmission in this population. The aim of this study was two-fold: 1) to evaluate the validity of the study cohorts in terms of health care utilization in relation to regional cohorts; 2) to describe the study cohorts in terms of health status and health care utilization after discharge.

**Methods:**

The project consist of two cohorts with data from patient records of geriatric in-hospital stays, health care utilization data from Stockholm Regional Healthcare Data Warehouse 6 months after discharge, socioeconomic data from Statistics Sweden. The 2012 cohort include 6710 patients and the 2016 cohort, 8091 patients; 64% are women, mean age is 84 (SD 8).

**Results:**

Mean days to first visit in primary care was 12 (23) and 10 (19) in the 2012 and 2016 cohort, respectively. Readmissions to hospital was 38% in 2012 and 39% in 2016. The validity of the study cohorts was evaluated by comparing them with regional cohorts. The study cohorts were comparable in most cases but there were some significant differences between the study cohorts and the regional cohorts, especially regarding amount and type of primary care.

**Conclusion:**

The study cohorts seem valid in terms of health care utilization compared to the regional cohorts regarding hospital care, but less so regarding primary care. This will be considered in the analyses and when interpreting data in future studies based on these study cohorts. Future studies will explore factors associated with health status and re-admissions in a population with multi-morbidity and disability.

## Background

The average life expectancy of people aged 65 years in the EU member states is 21.6 years for women and 18.2 for men (women 17.6–24 years; men 14.1–19.7 years). People in the Nordic countries live a greater number of healthy years without activity limitation compared to other parts of Europe [1]. However, people live longer with chronic conditions today and the prevalence of disability increases with age [2]. Multi-morbidity is common among older people [3, 4] and a study recently showed that a rapid development of multi-morbidity increases the risk of disability [5]. In addition, a poor social network and female sex increased the risk even further [5]. To cope with both multi-morbidity and disability, care coordination is important, and the organization of care is complex, requiring an inter-professional team approach to handle multi-morbidity and disability. Coordination for this group must exist between departments (in-patient and outpatient) as well as within a team in one department [6]. Lack of follow-up after discharge has been shown to increase the risk of readmissions [7] and discharge planning and care management has been shown to reduce readmissions [8, 9]. Identifying people with a high risk for hospital admissions are of importance [10].

It has been stated that a lack of coordination between in and outpatient care is common. However, there is a variety in how health care service is organised and relates to demands (urgency and illness severity in the population), and supply (care practices and processes) [11]. One British report concluded that some primary care areas in Britain that had integrated and developed their services for older people had lower rates of hospital admissions, reported as hospital bed use [12]. A report from the European Union (EU) showed that a significant proportion of emergency department visits were patients with problems not requiring emergency care. A higher number of people in Sweden reported that they visited emergency departments because primary care was not available, compared to the average in the EU [1]. However, there is an ongoing shift in Sweden, where more care shall be given closer to the patient in primary care as well in the patients’ homes [13]. In the EU member states, the proportion of inpatient and outpatient care, as well as for long-term care, varies to a large extent. However, the large differences in long-term care depend on differences in how care for older people and people with disabilities is organized and whether or not social care (both institutions and home care services) are included. The northern countries have a larger proportion of long-term care compared to the central and southern countries, which have larger proportions of informal care [1].

As stated above, care coordination and discharge planning are important to ensure high quality care for older people. However, the level of health care utilization among older people with multi-morbidity and disability has received little attention. This population is very common in geriatric departments in Sweden [14]. There is also a lack of knowledge at population level about the health status of geriatric patients in terms of diseases, medications, risks and disability. Therefore, a register-based research program has been initiated to increase knowledge of the health status of this population and to identify risk factors for readmission in geriatric patients. One cohort from 2012 and one from 2016 are used in the program and consist of data from the patients’ records during admission to three geriatric departments, health care utilization data after discharge, and socioeconomic data.

To determine the validity of the cohorts compared to the standard population, the aim of this specific study is two-fold: 1) to evaluate whether the study cohorts are comparable in terms of health care utilization to the regional cohorts using the regional cohorts as a standard population, and 2) to describe the study cohorts in terms of age, sex, health status and health care utilization after discharge.

## Methods

The design of the study is closed cohorts and based on registry data. We used a data set from 2012 to 2013 based on patients discharged from three geriatric departments in the Stockholm Region and a corresponding data set from 2016 to 2017 (study cohorts) to answer the overall research questions in the project. The data set consists of data from the patient records of geriatric hospital stays (index admission) and health care utilization for 6 months after discharge extracted from the Stockholm Regional Healthcare Data Warehouse (VAL), as well as socioeconomic data from Statistics Sweden (https://www.scb.se/). For an overview of data variables, see Table 1. To evaluate validity of health care utilization data, comparison data for the whole region was also retrieved from the VAL database (regional cohorts) and used as the standard population. Ethical permissions were approved by the Regional Ethical Board in Stockholm (Dnr 2013/1620–31/2; 2018/247–32).

**Table 1:** Data collection from different registers

Variables	Patient records	Health care consumption	Statistics Sweden
**Demographics**			
Age	x		
Sex	x		x
Marital status			x
Living alone/together			x
Education			x
Occupation			x
Income			x
Place of birth			x
**Diagnoses and drugs**			
Type of diagnoses	x	x	
Type of drugs	x		
**Physical examinations**			
Blood pressure	x		
Body temperature	x		
Saturation	x		
**Blood samples**			
Electrolytes	x		
Haemoglobin	x		
C-reactive protein	x		
Kidney function*	x		
**Risk screening**			
Malnutrition	x		
Fall	x		
Pressure ulcer	x		
**Physical function**			
ADL	x		
Mobility*	x		
**Care process:**			
Admitted from	x		
Discharged to	x		
*Hospital care*			
Length of stay	x	x	
Type of admission		x	
Time to admission		x	
Type of intervention		x	
*Primary care*			
Type of visit		x	
Time to visit		x	
Type of caregiver		x	
Type of intervention		x	

*Abbreviation*: *ADL* Activities of daily living, * = only cohort 2016

### Setting

The study was conducted in the region of Stockholm with 26 municipalities. In 2018, 2.3 million people resided in this region, of which approximately 390,000 were age 65 and older. The healthcare system consists of one university hospital, five acute care hospitals with emergency departments, and thirteen geriatric departments, In Stockholm, geriatric care is defined as care for patients in need of the competence of a geriatric team and not in anticipated need of the resources of the acute care hospital. Primary health care (PHC) consists of approximately 210 health care centres with GPs and district nurses as well as 60 rehabilitation clinics. There are also local emergency clinics with access to acute care appointments between the hours 800–2200. In this region, primary care is responsible for home health care, including home rehabilitation, for older people living at home. The municipalities are responsible for home care services for all older people and for nursing homes and residential care homes for older people. One goal for healthcare in the region is to increase the PHC including home healthcare to prevent visits to hospital-based emergency departments and readmissions to hospitals.

### Study cohorts

#### Study population

##### Inclusion criteria

All patients admitted in 2012 and 2016 were included.

##### Exclusions criteria

Patients who died during the index admission or were not living in Stockholm Region at the time of the index admission were excluded.

Data in the first study cohort consists of all admissions (*n* = 10,062) in 2012 at three geriatric departments, resulting in 6710 individual patients. Data in the second study cohort (2016) from the same geriatric departments includes 11,479 admissions resulting in 8091 individual patients. If a patient had more than one hospital admission to the geriatric departments during the data collection period, the last admission was used as the index admission in this study.

#### Data collection

Three sources were used to collect data (see Table 1):

- Existing and relevant information from the electronic patients’ records were extracted by the region (index admission).
- Data on health care utilization was retrieved from the VAL database up until 6 months after discharge from the index admission. All healthcare providers within the Stockholm Region – including both hospitals and outpatient departments (primary care) – are obliged to report the data in digital form. The VAL database covers 99% of all care in Stockholm.
- Data on socioeconomic factors was retrieved from Statistics Sweden, which is responsible for official statistics and for other government statistics in Sweden (cohort 2016).

##### Demographic data

- Age, sex, length of stay, education, living conditions, marital status, profession, income, place of birth (Continent).

##### Care process

- Length of stay, admitted from, discharged to.

##### Diagnoses and drugs

- Type (primary and secondary) and number of medical diagnoses.

- Prescribed drugs (continuous and pro re nata) at admission and at discharge.

##### Physical examinations

- Blood pressure, heart rate, saturation body temperature, height and weight.

##### Blood sample

- Haemoglobin, C-reactive protein (CRP), and electrolytes were taken when indicated.

- Creatinine was taken when indicated (2016). Estimated glomerular function rate (eGFR) was recorded to estimate kidney function and the eGFR is divided in normal, mild, moderate, severe, and end stage based on creatinine level, age, sex and weight [15].

##### Risk screening

- The Mini Nutritional Assessment was used to screen for malnutrition [16]. The instrument consists of five domains, such as weight loss, mobility and decreased food intake. It is based on scores between 0 and 14 points, where 0–7 is regarded as malnutrition, 8–11 is regarded as risk for malnutrition and 12–14 as normal nutritional status.

- The Norton scale was used to screen for risk of ulcer pressure [17]. The instrument consists of seven domains, such as mental status, intake of fluid and food, and mobility. It is based on scores between 7 and 28 points. A score of 20 and below is regarded as risk of ulcer pressure.

- The Downton Fall Risk Index to screen for fall risk [18]. The instrument consists of five domains, such as previous falls, gait security, and medications. It is based on scores between 0 and 11. Three or more points indicate an increased fall risk.

##### Physical function

- Basic activities of daily living (ADL) were measured with the Katz Index (cohort 2012) and the Barthel Index (cohort 2016) [19, 20]. The Katz Index consists of six activities in basic ADL, such as bathing, dressing, and toileting. Each activity is rated with three grades; independent, partly dependent and dependent. This was converted to an ordinal scale from 0 (independent) to 2 (dependent). A summa score from 0 to 12 was created based on the six activities, where 0 indicates total independence and 12 total dependency. The Barthel Index consists of 10 activities (adding, for example, grooming and ability to climb stairs), where each activity is rated as independent (15 points), partly dependent (10 points), or dependent (0 points). A total score of 100 points indicates total independence.

- Mobility was measured with the Rivermead Mobility Index (cohort 2016) [21]. The scale consists of 15 ambulation activities, such as getting in and out of bed, picking up objects from the floor, and ability to climb stairs. It is rated as ability (1 point) or non-ability (0 points). A total score of 15 points indicates independence in mobility.

##### Health care utilization after discharge

Data was retrieved up to six months after discharge from the index admission.

Hospital care process: Admissions to hospital with information on type of department, registered diagnoses and treatment, date of admission and discharge. Patients could in a few cases be readmitted the same day as discharge, either from home or by transfer between clinics. Only those readmitted from home was considered as a readmission.

Primary care process: Visits to primary care including information on setting (department, home care and/or home rehabilitation), type of visits (caregiver), date of visits, type of interventions (assessment or treatment).

### Regional cohorts

To analyse the comparability between the study cohorts and the region, aggregated health care utilization data was retrieved for the whole region from the VAL database and used as the standard population. The same inclusion and exclusion criteria were used, as well as health care utilization data as described above. There is an overlap of patients between the study and the regional cohorts, the study cohort makes up about 35% of the regional cohort.

### Statistical analyses

Descriptive analysis was performed with proportion, mean and standard deviation or median and interquartile range according to data level. Clinical data from several geriatric departments are generally time-consuming and difficult to collect, but data collected from a few departments and merged with socioeconomic data and other health records may provide effective and low-cost information. To validate the study cohorts, incidence rates (IR) of health events based on data from three geriatric departments in Stockholm (study cohort) were compared with incidence rates reported in the Stockholm Region VAL database (regional cohorts) [22]. A two-sided exact significance test “mid-P” calculation was used to compare two crude incidence rates between the regional cohorts and study cohorts for 2012 and 2016 (ecological comparison with aggregated data) [23]. These calculations determined the validity of the study cohorts; a significant value higher than 5% indicated that data are comparable and valid. If 1 is included in the estimated 95% confidence interval (CI) of the incidence rate ratio (IRR), then we assume that the study cohort is equal to the regional cohort. Therefore, 95% CI for all IRR are presented [24].

Registered main diagnoses were categorized according to the International Classification of Diseases (ICD)-10 chapters (https://icd.who.int/browse10/2016/en). Health status is described divided by sex. However, due to oversampling and multiple testing and, therefore, risk of type 1 error, statistical analyses were not conducted to evaluate differences between sexes or between study cohorts. Clinically relevant differences are described and discussed. The analyses were conducted in SPSS 26, SAS 9.4, Stata 14, StatDirect 3 and Excel 2016.

## Results

### Validity of the study cohorts

The crude incidence rate ratio analyses showed that there were some significant differences between the study cohorts and the regional cohorts, especially regarding primary care (see Tables 2 and 3). First contact with nurses after discharge was less in the study cohorts compared to regional cohorts, and the opposite was seen for physiotherapists and occupational therapists. There were also fewer home visits in the study cohorts compared to the regional cohorts (Table 2). Regarding readmission, it was more common to be admitted to an internal medicine department in the regional cohorts. The opposite was true for admission to a geriatric department (Table 3).

**Table 2:** Validity of study population. Data shows the six-month follow-up of primary care contacts after discharge

Variables	2012			2016		
	Study cohort ***n*** = 6710	Region cohort *n* = 20,029	Ecological comparison	Study cohort ***n*** = 8091	Region cohort ***n*** = 22,906	Ecological comparison
**First contact after discharge, n (%)** ^**b**^						
Physician	1242 (19)	3894 (19)	**0.95 (0.89–1.02)**	1390 (17)	4215 (18)	0.93 (0.88–0.99)
Nurse/District nurse	1776 (26)	5932 (30)	0.89 (0.85–0.94)	2112 (26)	6339 (28)	0.94 (0.90–0.99)
Occupational therapist	748 (11)	1728 (9)	1.29 (1.18–1.41)	1137 (14)	2453 (11)	1.31 (1.22–1.41)
Physiotherapist	662 (10)	1385 (7)	1.43 (1.30–1.57)	902 (11)	2158 (9)	1.18 (1.09–1.28)
Assistant nurse	389 (6)	1342 (7)	0.87 (0.77–0.97)	656 (8)	1859 (8)	**1.00 (0.91–1.09)**
Other	133 (2)	309 (2)	1.28 (1.04–1.58)	191 (2)	527 (2)	**1.03 (0.86–1.21)**
No visits	1655 (26)	5439 (27)	0.91 (0.86–0.96)	1703 (21)	5355 (23)	0.90 (0.85–0.95)
**Days to first visit, mean (sd)** ^**a**^	12 (23)	13 (24)	12.75 (12.46–13.03)	10 (19)	11 (21)	10.74 (10.51–10.97)
**Number of visits in the first six months after discharge, n (%)** ^**b**^	*n* = 187,096	*n* = 576,508		*n* = 292,613	*n* = 816,839	
Physician	31,125 (17)	85,114 (15)	1.13 (1.11–1.14)	38,322 (13)	97,850 (12)	1.09 (1.08–1.11)
Nurse/District nurse	78,143 (42)	261,515 (45)	0.92 (0.91–0.93)	102,877 (35)	301,739 (37)	0.95 (0.95–0.96)
Occupational therapist	9350 (5)	25,900 (4)	1.11 (1.09–1.14)	12,826 (4.3)	29,615 (3.6)	1.21 (1.18–1.23)
Physiotherapist	12,756 (7)	35,790 (6)	1.10 (1.08–1.12)	20,762 (7)	51,468 (6)	1.12 (1.11–1.14)
Assistant nurse	52,941 (28)	161,723 (28)	**1.01 (1.00–1.02)**	108,852 (37)	324,890 (40)	0.94 (0.93–0.94)
Dietician	1070 (0.6)	2352 (0.4)	1.40 (1.30–1.51)	2447 (0.8)	5796 (0.7)	1.17 (1.12–1.24)
Speech therapist	1227 (0.7)	2685 (0.5)	1.41 (1.32–1.51)	1275 (0.4)	3421 (0.4)	**1.04 (0.97–1.11)**
Social worker	430 (0.2)	1225 (0.2)	**1.08 (0.97–1.21)**	758 (0.25)	1842 (0.23)	1.15 (1.05–1.25)
Psychologist	54 (0.03)	204 (0.04)	**0.82 (0.59–1.11)**	49 (0.02)	218 (0.03)	0.63 (0.45–0.86)
**Type of visits after discharge in the first six months, n (%)** ^**b**^						
Department based	41,185 (22)	112,324 (19)	1.13 (1.12–1.14)	45,224 (15)	117,299 (14)	1.08 (1.06–1.09)
Home visits	142,826 (76)	456,765 (79)	0.93 (0.96–0.97)	230,357 (79)	658,322 (81)	0.98 (0.97–0.98)
Team visits	608 (0.3)	2251 (0.4)	0.83 (0.76–0.91)	10,083 (3.4)	21,291 (2.6)	1.32 (1.29–1.35)
Administrative work related to care	2477 (1)	5168 (0.9)	1.47 (1.41–1.55)	6949 (2.3)	19,927 (2.4)	**0.97 (0.95–1.00)**

*Abbreviations*: *n* number, *m* mean, *sd* standard deviation, Ecological comparison = comparing aggregated data on the group level

Bold style indicate a two-sided exact non-significance, tests usually called “midp” calculation, to control for differences of crude incidence rate comparing Region and Study cohorts at year 2012 and 2016

a: Performs t-tests on the equality of means assuming a hypothesis that combined mean of study population and region population is equal

b: Incidence rate ratio (95% CI)

**Table 3:** Validity of study population. Data shows the six-month follow-up of hospital care contacts after discharge

Variables	2012			2016		
	Study cohort *n* = 6710	Region cohort *n* = 20,029	Ecological comparison	Study cohort *n* = 8091	Region cohort *n* = 22,906	Ecological comparison
**Patients re-admitted, n (%)** ^**b**^	2542 (38)	7194 (36)	1.05 (1.01–1.10)	3145 (39)	8144 (36)	1.09 (1.05–1.14)
**Number of admissions of the readmitted, m (sd)** ^**a**^	1.9 (1.5)	1.9 (1.45)	1.9 (1.88–1.92)	2.1 (1.6)	1.97 (1.5)	2.0 (1.99–2.02)
**Days to first re-admission, m (sd)** ^**a**^	60 (55)	61 (55)	60.52 (59.60–61.43)	59 (54)	59 (54)	59 (58.40–59.60)
**Admitting departments at first visit, n (%)** ^**b**^	*n* = 2542	*n* = 7194		*n* = 3145	*n* = 8144	
Internal medicine	757 (30)	2422 (34)	0.88 (0.83–0.95)	934 (30)	2729 (34)	0.89 (0.82–95)
Geriatric	413 (16)	896 (13)	1.30 (1.18–1.44)	512 (16)	974 (12)	1.36 (1.22–1.52)
Surgery	326 (13)	698 (10)	1.32 (1.16–1.51)	432 (14)	807 (10)	1.39 (1.23–1.56)
Cardiology	282 (11)	714 (10)	**1.12 (0.97–1.28)**	289 (9)	768 (9)	**0.97 (0.85–1.12)**
Orthopedic	220 (9)	572 (8)	**1.09 (0.93–1.27)**	235 (7)	586 (7)	**1.04 (0.89–1.21)**
Infection	80 (3)	258 (4)	**0.88 (0.67–1.13)**	112 (4)	236 (3)	**1.23 (0.97–1.55)**
Neurology	40 (2)	178 (2)	0.64 (0.44–0.90)	120 (4)	327 (4)	**0.95 (0.76–1.17)**
Urology	77 (3)	204 (3)	**1.07 (0.81–1.39)**	85 (3)	254 (3)	**0.86 (0.67–1.11)**
Kidney	41 (2)	74 (1)	1.57 (1.04–2.33)	69 (2)	110 (1)	1.62 (1.18–2.22)
Psychiatry	38 (1)	107 (1)	**1.01 (0.68–1.47)**	64 (2)	170 (1)	**0.97 (0.72–1.31)**
Other	268 (11)	1071 (15)	0.71 (0.62–0.81)	295 (9)	1183 (15)	0.64 (0.57–0.73)
**Main diagnoses at first visit, n (%)** ^**b**^	*n* = 2522	*n* = 7136		*n* = 3136	*n* = 8119	
Certain infectious and parasitic diseases	126 (5)	330 (5)	**1.08 (0.87–1.33)**	133 (4)	376 (5)	**0.92 (0.75–1.12)**
Neoplasms	214 (8)	624 (9)	**0.97 (0.83–1.14)**	207 (7)	660 (8)	0.81 (0.69–0.95)
Blood and blood-forming organs	37 (1)	104 (1)	**1.01 (0.67–1.48)**	40 (1)	112 (1)	**0.92 (0.63–1.34)**
Endocrine, nutritional and metabolic	63 (3)	186 (3)	**0.96 (0.71–1.28)**	89 (3)	222 (3)	**1.04 (0.80–1.33)**
Mental and behavioral	110 (4)	263 (4)	**1.18 (0.94–1.48)**	143 (5)	328 (4)	**1.13 (0.92–1.38)**
Nervous system	96 (4)	233 (3)	**1.17 (0.91–1.48)**	109 (3)	247 (3)	**1.14 (0.90–1.44)**
Eye and adnexa	11 (0.5)	31 (0.5)	**1.00 (0.46–2.05)**	11 (0.5)	35 (0.5)	**0.81 (0.37–1.64)**
Ear and mastoid process	8 (0.5)	20 (0.5)	**1.13 (0.43–2.68)**	8 (0.5)	18 (0.5)	**1.15 (0.43–2.78)**
Circulatory system	507 (20)	1524 (21)	**0.94 (0.85–1.04)**	627 (20)	1638 (20)	**0.99 (0.90–1.09)**
Respiratory system	323 (13)	896 (13)	**1.02 (0.90–1.16)**	434 (14)	1181 (15)	**0.95 (0.85–1.06)**
Digestive system	172 (7)	517 (7)	**0.94 (0.79–1.12)**	210 (7)	595 (7)	**0.91 (0.78–1.07)**
Skin and subcutaneous tissue	31 (1)	93 (1)	**0.94 (0.61–1.43)**	30 (1)	95 (1)	**0.82 (0.52–1.24)**
Musculoskeletal system tissue	110 (4)	325 (5)	**0.96 (0.76–1.19)**	167 (5)	380 (5)	**1.14 (0.94–1.37)**
Genitourinary system	165 (7)	476 (7)	**0.98 (0.82–1.17)**	226 (7)	560 (7)	**1.04 (0.89–1.22)**
Congenital mal/deformations	2	3	–	0	0	–
Symptoms abnormal departmental findings	194 (8)	568 (8)	**0.97 (0.82–1.14)**	272 (9)	671 (8)	**1.05 (0.91–1.21)**
Injury, poisoning	283 (11)	760 (11)	**1.05 (0.92–1.21)**	360 (11)	803 (10)	1.16 (1.02–1.32)
External causes of morbidity/mortality	0	0	–	0	0	–
Factors influencing health status	70 (3)	183 (3)	**1.08 (0.81–1.143)**	70 (2)	198 (2)	**0.92 (0.69–1.121)**
Codes for special purposes	0	0	–	0	3	–
**Visits to emergency departments**	*n* = 3264	*n* = 14,328		*n* = 3968	*n* = 15,011	
No admissions (range)	1959 (1–16)	8388 (1–39)	**0.98 (0.94–1.01)**	1980 (1–56)	7929 (1–56)	**0.94 (0.90–1.00)**
Admissions (range)	1305 (1–9)	5940 (1–9)	**0.94 (0.89–1.00)**	1988 (1–9)	7082 (1–11)	1.06 (1.01–1.12)

*Abbreviations*: *n* number, *m* mean, *sd* standard deviation, *ns* non-significant, *ECR* Emergency, ^a^ Categorized according to the ICD-10 chapters, Ecological comparison = comparing aggregated data on the group level

Bold style indicates a two-sided exact non-significance, tests usually called “mid-P” calculation, to control for differences of crude incidence rate comparing Region and Study cohorts at year 2012 and 2016

a: Performs t-tests on the equality of means assuming a hypothesis that combined mean of study population and region population is equal

b: Incidence rate ratio (95% CI)

### Demographic characteristics and health status

Baseline characteristics from the index admission are shown in Table 4. The mean age was 84 (SD 8) in both cohorts and there were 64% women in the 2012 and 63% in the 2016 cohort. The women were older in both study cohorts. In the 2016 study cohort, the majority of women lived alone and had a lower educational level than the men.

**Table 4:** Baseline characteristics at the index admission in the study cohorts

Variables	2012			2016		
	Total *n* = 6710	Women ***n*** = 4310	Men ***n*** = 2400	Total ***n*** = 8109	Women ***n*** = 5070	Men ***n*** = 3021
**Age**, mean (sd)	84 (7.8)	84.8 (7.7)	82.9 (7.9)	83.5 (8.2)	84.3 (8.1)	82.1 (8.1)
**Civil status**, n (%)				*n* = 8076	*n* = 5061	*n* = 3015
Married	–	–	–	2321 (29)	919 (18)	1399 (46)
Unmarried	–	–	–	834 (10)	480 (9)	354 (12)
Divorced	–	–	–	1672 (21)	1103 (22)	569 (19)
Widow, Widower	–	–	–	3260 (40)	2559 (51)	696 (23)
				*n* = 8071	*n* = 5059	*n* = 3012
Living alone	–	–	–	4903 (61)	3565 (70)	1338 (44)
**Education**, n (%)				*n* = 7840	*n* = 4920	*n* = 2920
Primary	–	–	–	1723 (22)	1152 (23)	571 (20)
Lower secondary	–	–	–	998 (13)	792 (16)	206 (7)
Upper secondary	–	–	–	2840 (36)	1692 (34)	1148 (39)
Post-secondary	–	–	–	1523 (19)	960 (20)	563 (19)
Higher post-secondary	–	–	–	756 (10)	324 (7)	432 (15)
**Continent of birth**, n (%)				*n* = 8080	*n* = 5064	*n* = 3016
Sweden	–	–	–	6677 (83)	4134 (82)	2543 (84)
Other Nordic countries	–	–	–	602 (7)	442 (9)	159 (5)
Other Europe	–	–	–	532 (7)	330 (6)	202 (7)
Outside Europe	–	–	–	270 (3)	158 (3)	112 (4)
**Number of diagnoses**, m (sd)	4.1 (1.7)	4 (1.7)	4.3 (1.8)	4.6 (1.8)	4.5 (1.8)	4.9 (1.9)
**Number of continuous medications**, m (sd)	6.7 (3.8)	6.8 (3.8)	6.4 (3.8)	8.6 (4.2)	8.6 (4.2)	8.6 (4.1)
**Care processes** n (%)**,** *Admitted from:*						
Other clinic/hospital	3423 (51)	2196 (51)	1227 (51)	5404 (67)	3333 (66)	2071 (69)
Home	3073 (46)	1984 (46)	1089 (45)	2631 (32)	1706 (34)	925 (30)
Nursing home	214 (3)	130 (3)	84 (4)	56 (1)	31 (1)	25 (1)
*Discharged to:*	*n* = 6708	*n* = 4310	*n* = 2398			
Other clinic/hospital	346 (5)	224 (5)	122 (5)	505 (6)	284 (6)	221 (7)
Home	4728 (71)	3063 (71)	1665 (69)	6215 (77)	3844 (78)	2271 (75)
Nursing home	1355 (20)	880 (21)	475 (20)	1215 (15)	775 (15)	440 (15)
Deceased	279 (4)	143 (3)	136 (6)	151 (2)	64 (1)	87 (3)
*Length of stay, m(sd)*	10.1 (7.2)	10 (6.6)	10.3 (8.1)	9.1 (5.7)	9.1 (5.5)	9.1 (6)
**Nutrition** (MNA-SF), n (%)	*n* = 6573	*n* = 4234	*n* = 2339	*n* = 7926	*n* = 4963	*n* = 2963
Malnutrition	2025 (31)	1335 (32)	690 (29)	2266 (29)	1406 (28)	860 (29)
Risk of malnutrition	3371 (51)	2160 (51)	1211 (52)	4289 (54)	2727 (55)	1562 (53)
Normal	1177 (18)	739 (17)	438 (19)	1371 (17)	830 (17)	541 (18)
**Pressure ulcer** (Norton), n (%)	*n* = 6588	*n* = 4240	*n* = 2348	*n* = 7982	*n* = 5004	*n* = 2978
Risk	2108 (32)	1368 (32)	740 (32)	2144 (27)	1340 (27)	804 (27)
**Fall risk** (Downton), n (%)	*n* = 6654	*n* = 4271	*n* = 2383	*n* = 7997	*n* = 5001	*n* = 2986
Risk	5330 (80)	3416 (80)	1914 (80)	6854 (86)	4291 (86)	2563 (86)
**Kidney function,** n (%)				*n* = 3651	*n* = 2252	*n* = 1399
Normal	–	–	–	273 (7)	146 (6)	127 (9)
Mild	–	–	–	1766 (48)	1110 (49)	656 (47)
Moderate	–	–	–	1110 (30)	705 (31)	405 (29)
Severe	–	–	–	485 (13)	284 (13)	201 (14)
End stage	–	–	–	17 (2)	7 (1)	10 (1)
**Laboratory data**, m (sd)						
	*n* = 3086	*n* = 1910	*n* = 1176	*n* = 3651	*n* = 2252	*n* = 1399
Creatinine, mg/dL	99 (63)	89 (52)	116 (74)	98 (59)	88 (46)	116 (73)
	*n* = 3037	*n* = 1881	*n* = 1156	*n* = 3561	*n* = 2208	*n* = 1353
Sodium, mEq/L	139 (4)	138.6 (4)	139.1 (4)	139 (4)	138.5 (3.9)	139 (4.2)
	*n* = 130	*n* = 79	*n* = 51	*n* = 3618	*n* = 2241	*n* = 1377
Potassium, mg/dL	4 (0.4)	4 (4)	4 (2)	4 (0.5)	4 (0.4)	4 (0.5)
	*n* = 3019	*n* = 1855	*N* = 1164	*n* = 3590	*n* = 2179	*n* = 1411
Hemoglobin, g/L	117 (16)	116 (15)	118 (17)	115 (15)	114 (14)	116 (17)
	*n* = 1806	*n* = 1047	*n* = 759	*n* = 3422	*n* = 2095	*n* = 1327
C-reactive protein, mg/L	47 (52)	43 (47)	52 (57)	42 (44)	39 (39)	47 (51)
**Physical examinations**, m (sd)	*n* = 6033	*n* = 3874	*n* = 2159	*n* = 7552	*n* = 4737	*n* = 2815
Blood pressure, Systolic	128 (20)	129 (20)	125 (19)	125 (17)	127 (17)	123 (17
	*n* = 6030	*n* = 3872	*n* = 2158	*n* = 7548	*n* = 4734	*n* = 2814
Blood pressure, Diastolic	69 (11)	69 (11)	69 (11)	68 (10)	68 (10)	68 (10)
	*n* = 4435	*n* = 2826	*n* = 1609	*n* = 6058	*n* = 3821	*n* = 2237
Saturation	94 (4)	94 (3.8)	98 (4.1)	95 (3)	95 (3.3)	96 (3.4)
	*n* = 726	*n* = 450	*n* = 276	*n* = 7554	*n* = 4722	*n* = 2832
Body temperature	36.5 (0.6)	37 (0.5)	36 (0.6)	36.7 (0.5)	36.7 (0.48)	36.6 (0.51)
**Physical function**						
	*n* = 6617	*n* = 4254	*n* = 2363			
Katz Index, md (q1-q3)	6 (2–10)	6 (2–10)	6 (2–10)	–	–	–
				*n* = 5092	*n* = 3284	*n* = 1808
Barthel index, md (q1-q3)	–	–	–	70 (45–85)	70 (45–85)	70 (45–85)
				*n* = 5112	*n* = 3252	*n* = 1860
Rivermead mobility index, md (q1-q3)	–	–	–	7 (4–9)	7 (4–9)	7 (4–10)

*Abbreviations*: *n* number, *m* mean, *sd* standard deviation, *ns* non-significant,

*MNA-SF* Mini Nutritional Assessment-Short Form, *mg* milligrams, *dL* deciliter, *mEq* milliequivalent, *g* grams, *L* Liter

Regarding the health status of the patients, 80% were at risk of falling in 2012 and 85% in 2016. Half of them were at risk for malnutrition and one third were identified as malnourished in both cohorts. One-third were at risk of a pressure ulcer in the 2012 cohort. In the 2016 study cohort, the proportion was 27%. The data on physical examinations and blood chemistry were within reference values (except CRP) in both study cohorts. Men had higher creatinine and CRP levels than the women. Data on physical function revealed that more than half of the patients were independent in more than half of the activities, with similar values in women and men.

When admitted to the geriatric departments in 2012, the patients on average had 4.1 (1.7) diagnoses and were prescribed 6.7 (3.8) continuous medications. In the 2016 study cohort the mean diagnoses were 4.6 (1.8) and the prescriptions of continuous medications were 8.6 (4.2).

Regarding the care processes related to the index admissions, 46% were admitted from home in the 2012 cohort, while the corresponding number for 2016 was 34%. Discharge to home was 71% in the 2012 cohort and 78% in 2016. Discharge to nursing homes was 21% in 2012 and 15% in 2016.

### Health care utilization after discharge

Mean days to first contact with primary care after discharge was 12 (23) days in the 2012 study cohort and 10 (19) in 2016 study cohort. The most common first professions the patients had contact with after discharge were registered nurses (26%) and physicians (19% in 2012 and 18% in 2016). The vast majority of types of visits were home visits (Table 2).

There were few primary care intervention registrations (data not shown in tables). The three most common interventions in 2012 study cohort were home rehabilitation (*n* = 14,910), prescriptions for assistive devices (*n* = 2051) and conferences about a patient (*n* = 1346). The three most common in the 2016 study cohort were home rehabilitation (*n* = 31,968), palliative care (*n* = 5150) and muscle function/strength training (*n* = 4926). An intervention that was only registered in the 2016 study cohort was a pharmaceutical review, which was registered 4028 times. No other interventions were registered in a systematic manner, so they were trackable in the VAL database.

**Fig. 1:**
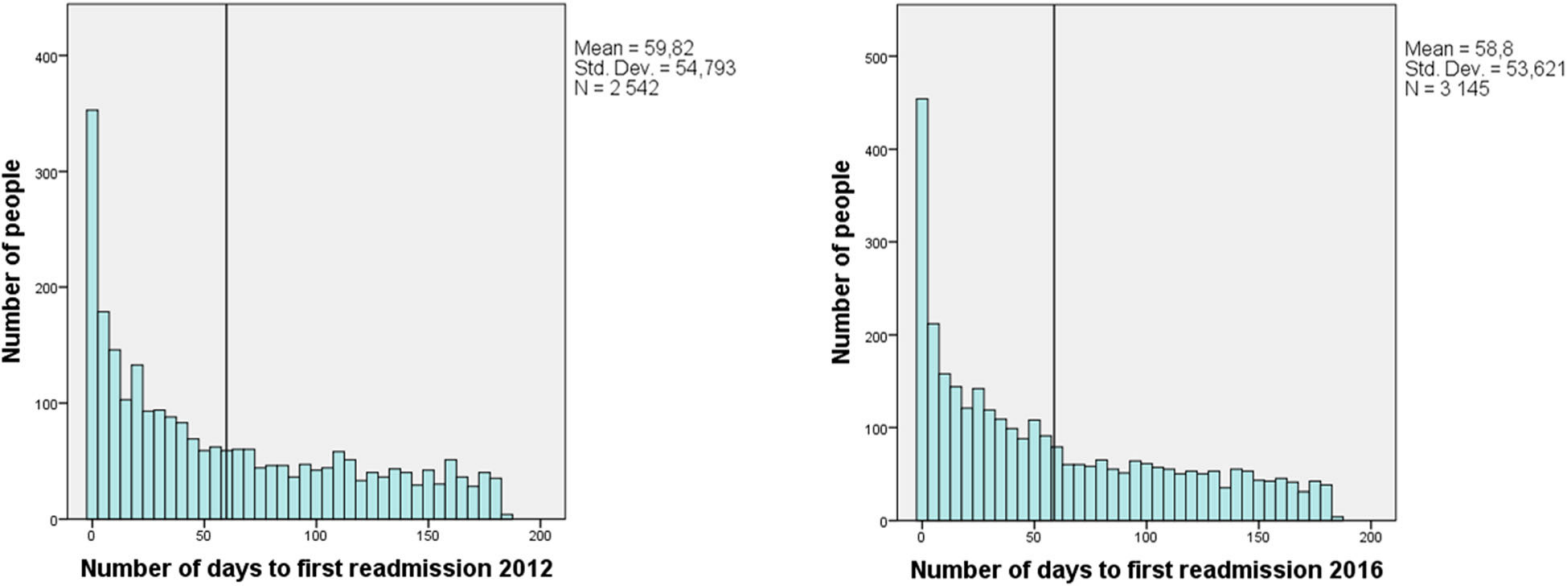
**a)** Number of days to first readmission 2012 **b)** Number of days to first readmission 2016

Amount and type of hospital care after discharge are shown in Table 3. Thirty-eight percent were re-admitted to the hospital within 6 months during 2012 and 39% during 2016. Figure 1a and b show the number of days to first hospital re-admission for the respective cohorts. The most common admitting department was Internal Medicine in both cohorts and the most common main diagnoses were within the circulatory system.

Interventions during the hospital stays were categorized in surgical and non-surgical interventions (data not shown in tables). In the 2012 study cohort, 827 (47%) surgical and 919 (53%) non-surgical interventions were registered. Corresponding numbers for the 2016 study cohort were 1072 (33%) surgical and 2202 (67%) non-surgical interventions.

## Discussion

The results showed that the participants had a high number of diseases, risks and disability levels: there were also some differences related to sex at the index admission in both study cohorts. We found some differences in incidence rate ratios between the study cohorts and the regional cohorts, especially regarding primary care, though the proportional differences were low.

### Validity of the study cohorts

There were some statistically significant differences between the study cohorts and the regional cohorts, especially regarding primary care. However, the proportional differences were small, which was confirmed by the narrow confidence intervals, and by the fact that the IRR in most cases was close to 1. However, some differences, such as differences in first contact and type of visits, might be explained by the fact that there are a number of PHC spread over the region and approximately half of them are run by private companies. Even though their assignment is equal and directed by the healthcare authorities, the interpretation and how they decide to prioritize might vary. A variety of how care is delivered has been suggested to be related to several factors such as variation in patient groups and patient preferences, cultural and professional norms, professional uncertainty about what to do and organizational design [25]. There were very few statistical differences regarding hospital care. There are five acute care hospitals in Stockholm Region, which might explain the lesser diversity. The differences regarding the admitting department might be related to geographical distances between the acute care hospitals and the geriatric departments as well as the fact that some geriatric departments do not have access to other departments for various assessments such as radiography and laboratories for chemistry analyses during evening, nights, and weekends.

### Demographic characteristics and health status

The socioeconomic data in the 2016 cohort showed several differences between the sexes. The women were less educated, but this was anticipated since women born in the first half of the twentieth century had little access to higher education [26]. More women than men were widowed and lived alone which is also expected and similar to the whole population in these age groups [27].

There were also some sex differences at the index admission in both cohorts regarding health issues. However, the differences were small and not deemed to be of clinical relevance. There were no differences in kidney function, but the differences in creatinine levels might be explained by the fact that men have a higher muscle mass than women. The elevated levels of CRP might be due to that CRP only was measured when clinically indicated, most often to monitor the course of an infection. Also, men often have more illnesses than women, which can explain the higher CRP values in men [28]. The results for ADL and mobility levels were similar, which is opposite to population-based studies where women usually have greater levels of disability compared to men [29, 30]. However, the contradictory result might be explained by the fact that the individuals in our cohorts have been admitted to a geriatric department in need of care and rehabilitation and, therefore, might have a higher and a more equal level of disability.

The numbers of diagnoses were somewhat smaller compared to a European multi-centre study where data was retrieved from primary care (General Practitioners) [31]. This difference might be due to our data being based on patient record data at discharge where physicians register those diagnoses relevant for the hospital stay. The results regarding the number of continuous medications in our study were lower compared to the same study [31], but the same as another Swedish study [32]. Geriatricians might be more prone to reducing the number of medications if possible, which might explain the lower number in our study [33]. However, there are contradictory results in a French study showing the opposite [34]. Nevertheless, setting and context might explain the different study results.

Regarding the care processes related to the index admissions, fewer people were admitted directly from home in the 2016 cohort, despite regional authorities trying to implement direct admissions to geriatric departments during this period to decrease the rate of older people going through the emergency departments, unless needed. One reason for the implementation of this guideline being unsuccessful may have been that several geriatric departments lack access to radiography and laboratory services 24/7 as described above.

### Health care utilization after discharge

The number of home and team visits from primary care were higher in 2016 compared with 2012. This might be due to the fact that authorities during this period directed rehabilitation clinics in primary care to prioritize home rehabilitation. When receiving a referral due to a hospital discharge, the home rehabilitation teams are obliged to do a first home visit within 24 h on weekdays. Another reason could be that the number of people discharged to nursing homes was lower in 2016, which might have led to an increased level of home visits by nurses and assistant nurses. The number of visits due to palliative care also increased in 2016 compared with 2012, indicating that older people are living at home at the end of life. The number of nursing home beds in the Stockholm Region has decreased in the last decade due to implementation of the policy “aging in place,” leading to more older people with complex care needs living at home [13, 35]. The larger number of visits by assistant nurses might reflect that older people living at home have basic nursing needs that the assistant nurses can handle, but it could also demonstrate the difficulties in recruiting and keeping registered nurses, a difficulty that has increased in Sweden in the last 10 years [36]. Another reason could be that during this period, most municipalities declined delegation to their social home care staff for administration of medication to their clients. This task was therefore moved to assistant nurses within home health care services run by the PHC.

Registration of interventions in the VAL database is low and is mainly reflected by how the reimbursement system is organized. The rehabilitation clinics are paid by number of visits and length of visits, which are registered in the database. Registration of interventions by physicians and nurses are not required for reimbursement. However, pharmaceutical reviews were registered to a larger extent in the 2016 cohort, which is probably due to PHC being reimbursed for implementing this intervention.

A previous report from the EU showed that older people in Sweden visited emergency departments more because primary care was not available, compared to the average in the EU [1]. One goal for the Stockholm Region has been to increase the number of healthcare centres and rehabilitation centres to provide the population, particularly the older population, access to healthcare to decrease the visits to emergency departments and hospitals [13]. According to our findings, even if the number of visits to the PHC did increase in 2016 compared with 2012, the percentage of visits to emergency departments and readmissions to the hospital did not decrease when comparing 2016 with 2012. There is a need to examine why this strategy has not been successful and what measures are needed to decrease the visits by older people to emergency departments and readmissions to hospital.

This geriatric population has a high burden of diseases, risks and disability levels, and the extent of health care utilization is large. This requires coordination between out of and in-hospital care in order to provide the best possible care. The fact the several interventions during this period have not resulted in expected changes is important to take into considerations by policy makers, stakeholders and authorities when re-organizing and re-structuring care. Increased knowledge of factors related to implementing reforms is vital in order to reach a successful result [37].

### Strengths and limitations

This study is based on registry data from several sources, which is a strength. However, data from the index admission consists of standardized documentation only, which limits information about the patients’ health status during hospitalization. Other major limitations include several missing values regarding laboratory data since these were only taken when indicated. Data on physical function was retrieved at discharge and especially in the 2016 cohort. There are missing data, which is a limitation. According to plan the patients shall be assessed both at admission and discharge but time restraints and short length of stay might explain why this was not always done. In addition, we do not know how the individuals’ health status developed over time after discharge. Another limitation is that we do not have baseline data of the patients’ health status in the regional cohort, since these data were retrieved from the VAL-data base that only collect and store information related to reimbursements. Also, we do not have information about the level of informal support for those living alone or those living with someone. As stated in the statistical analyses section, other limitations refer to multiple testing leading to a risk of type 1 error. Therefore, a confidence interval has been presented to let the readers themselves interpret the results [38]. Important strengths are the large sample sizes in both study cohorts, that individuals were followed over time, and the ability to combine data from different sources.

## Conclusion

The study cohorts seem valid compared to the regional cohorts in terms of health care utilization, especially regarding hospital care, but less so regarding primary care. This will be considered in the analyses as well as when interpreting data in future studies based on these study cohorts. The number of home visits, team visits and visits by assistant nurses was higher in the 2016 study cohort. The results regarding hospital care were similar in both study cohorts. Future studies using the database will explore factors related to health status, access to primary care, and investigating associations with visits to emergency departments and hospital readmissions, which could be preventable or avoidable. These results may be used as a base to develop reforms to improve care processes and health care for a vulnerable population.

## Data Availability

Data is not publicly available, but available upon request. Requests for access to the data can be put to our Research Data Office (rdo@ki.se) at Karolinska Institutet and will be handled according to the relevant legislation. This will require a data processing agreement or similar with the recipient of the data.
